# Emotional responses to auditory hierarchical structures is shaped by bodily sensations and listeners’ sensory traits

**DOI:** 10.3389/fpsyg.2025.1599430

**Published:** 2025-07-08

**Authors:** Maiko Minatoya, Tatsuya Daikoku, Yasuo Kuniyoshi

**Affiliations:** Graduate School of Information Science and Technology, The University of Tokyo, Tokyo, Japan

**Keywords:** auditory-induced emotion, emotional reaction, global/local processing, auditory syntactic patterns, sensory sensitivity, sensory profile, bodily sensations, emotion regulation

## Abstract

Emotional responses to auditory stimuli are a common part of everyday life. However, for some individuals, these auditory-induced emotions can become distressing enough to interfere with daily functioning. Despite the prevalence of these experiences, the mechanisms underlying auditory-induced emotional responses remain only partially understood. Previous research has identified several contributing factors, including features of the auditory stimuli, listener traits, and bodily sensations triggered by the stimuli. However, prior studies have primarily focused on the acoustic features of auditory stimuli, leaving the role of syntactic features largely unexplored. This study focuses specifically on hierarchical syntactic structures, examining how they influence emotional experiences in conjunction with listener traits and bodily sensations. We conducted an online experiment in which 715 participants listened to 26 sound sequences, each of which systematically varied in hierarchical syntactic structure. The sequences were generated by combining three types of local pitch movement with three types of global pitch movement, each presented in ascending and descending pitch directions, resulting in nine levels of combined complexity. Participants rated the emotional valence and arousal of each sequence and indicated any bodily sensations they experienced using a bodily map. Measures of sensory processing patterns were also collected. Results showed that emotional valence was associated with the complex interplay of moderate syntactic complexity (“not too simple, not too complex”), sensory sensitivity, and upper torso sensations. These findings contribute to existing research by identifying syntactic features that shape auditory-induced emotional experiences and by demonstrating the association between bodily sensations and emotional experience.

## 1 Introduction

How do emotions arise when we hear sounds? Feeling emotions as a response to sounds and sound sequences is a common part of our daily lives. We may feel scared by loud thunder, soothed by birds’ chirping, or energized by rock music. Yet, how sound sequences evoke specific emotional experiences is still not completely clear.

Although the mechanism of auditory-induced emotion is not clear, some of these emotions may be strong enough to become obstacles to daily life. Many individuals with sensory processing disorders (SPDs) suffer from negative emotional and behavioral responses induced by sensory input (Passarello et al., 2022). SPDs are linked to autism spectrum disorder (ASD), and it is known that many individuals with ASD show averse emotional and behavioral responses to auditory stimuli (Chang et al., 2012; Fernández-Andrés et al., 2015). Thus, understanding the mechanism of auditory-induced emotion is not only a theoretically, but also a clinically important question.

Various frameworks have been proposed to explain the mechanism of emotional experience induced by auditory stimuli, including widely accepted frameworks proposed by Scherer and Zentner (2001) and Juslin and Västfjäll (2008). Such frameworks generally agree that experienced (felt) emotion is a multiplicative function which consists of several factors including stimulus features (acoustic and syntactic features of the auditory stimuli), listener features (the listener’s musical experience, stable disposition such as personality, and transient state such as mood), and bodily experiences (physiological responses to the stimuli and the subjective experience of those responses).

Previous studies on stimulus features have revealed the acoustic features that relate to felt valence (pleasantness) and arousal (emotion activation). Gomez and Danuser (2007) found that mode (major vs. minor), harmonic complexity, and rhythmic articulation (e.g., staccato vs. legato) best predicted pleasantness, with major mode, simpler harmony, and staccato articulation correlating positively with higher pleasantness, respectively. In contrast, faster tempo, greater accentuation (e.g., marcato), and more staccato articulation were positively correlated with higher arousal, respectively. Coutinho and Cangelosi (2011) found positive linear correlations between valence and both pitch level and tempo—valence was higher in musical segments with higher pitch and faster tempi. They also found positive linear correlations between arousal and loudness, tempo, timbre, and pitch level—arousal was higher in segments with greater loudness, faster tempi, higher pitch, and sharper sounds. Jaquet et al. (2014) found that lower pitch level was associated with more negative valence and higher arousal by using piano excerpts that were systematically varied in pitch level. Furthermore, they found that gender moderated the effects of pitch level on both valence and arousal: the positive association between pitch and valence was stronger in women, while the negative association between pitch and arousal was observed only in men. They also observed that the effect of pitch level on valence was influenced by other musical features, such as tempo and mode.

Previous studies on listener features have revealed that certain listener traits are strongly associated with auditory-induced emotion. Liljeström et al. (2013) found that listeners with higher openness to experience tended to experience more intense and positive emotions (such as happiness and pleasure). Gerstgrasser et al. (2023) revealed that musical expertise, listeners’ personal traits (such as openness to experience), and current mood state are associated with enhanced intensity and differentiation (granularity) of the emotional experience. Sakka and Juslin (2018) suggested that severely depressed listeners tend to experience less happiness when listening to music that typically evokes happy memories. Kreutz et al. (2008) found a correlation between absorption traits and emotional arousal. Rawlings and Leow (2008) showed that psychoticism was associated with positive emotional responses to unpleasant music. Chang et al. (2012) pointed out that over- and under-responsiveness to auditory stimuli are related to problematic emotional and behavioral responses.

Studies have also investigated how bodily experiences—particularly physiological responses—contribute to emotional responses to auditory stimuli. For example, Dibben (2004) demonstrated that listening to music while in a physiologically aroused state enhances the intensity of the emotional experience. Coutinho and Cangelosi (2011) showed that emotional responses to music could be more accurately predicted by incorporating physiological measures such as skin conductance and heart rate. Gomez and Danuser (2007) found that fast, accentuated, and staccato music—stimulus features that were negatively associated with valence and positively associated with arousal—elicited increases in breathing rate, skin conductance, and heart rate.

Beyond physiological responses, researchers have also explored the role of bodily sensations, defined as the subjective experience of physiological responses, in shaping emotional experiences of auditory stimuli. Notably, many findings point to interactions between bodily sensations, physiological responses, and stimulus features. For example, Liljeström et al. (2013) observed a high level of self-reported emotion intensity as well as a high level of bodily arousal (measured by skin conductance and heart rate) when participants listened to self-chosen music. Putkinen et al. (2024) elucidated that acoustic features were linked with bodily sensations and emotion ratings across Western and East Asian cultures. Daikoku et al. (2024) provided insight into how musical expectation shapes emotional and physiological responses and revealed that chord progressions that shifted from low uncertainty and low surprise to low uncertainty and high surprise elicited cardiac sensations, bodily sensations in the heart area, which were associated with positive valence.

These findings have provided insight into the contributions of stimulus features, listener features, and bodily experiences to auditory-induced emotion. However, while stimulus features encompass both acoustic and syntactic features, prior research has primarily focused on acoustic features, leaving the study of syntactic features—organization of discrete structural elements into structured sequences (Asano and Boeckx, 2015)—largely unexplored.

Among such syntactic features are hierarchical syntactic structures—how elements of auditory stimuli are organized by local and nested global relationships (Koelsch et al., 2013). This structural complexity is a key component of human cognition in both music and language (Koelsch et al., 2013).

Previous research on hierarchical syntactic structures has been mainly focused on cognitive processing of such structures. Some of the main findings in the auditory modality include the precedence of global processing over local processing–changes in global pitch patterns (e.g., an overall rising pitch contour across a melody) being detected faster than local pitch patterns (e.g., a linear falling pattern within a shorter group of notes) (Sanders and Poeppel, 2007; List et al., 2007)–and the association between specific listener features, such as musical expertise and autism spectrum disorder, and enhanced local processing (Ouimet et al., 2012; Susini et al., 2020; Mottron et al., 2000).

In terms of the relationship between hierarchical syntactic structures and emotional experience, research is very limited. A few studies investigated the relationship in the visual modality and have suggested a bidirectional relationship between global processing and stronger happiness (Ji et al., 2019; Gasper and Clore, 2002). However, to the best of our knowledge, there are currently no studies that have investigated this relationship in the auditory modality. Moreover, there is no research on how bodily sensations affect emotions induced by hierarchical syntactic structures.

To address this gap, the current study investigated how the hierarchical syntactic structure of auditory stimuli influences emotional experience in conjunction with listener features and bodily sensations. To this end, we conducted an experiment where we created sound sequences that systematically varied in the complexity of the hierarchical structure and examined the subjective emotional and bodily response to those sequences. We hypothesized that variations in structural complexity will elicit distinct emotional responses in individuals with specific sensory processing patterns. Moreover, we predicted that bodily sensations would amplify those emotional responses, based on previous findings that bodily sensations enhanced emotion prediction (Coutinho and Cangelosi, 2011).

## 2 Materials and methods

### 2.1 Participants

715 Japanese adults (female = 357, male = 356, other = 2; mean age = 35.61 years, SD = 8.88) participated in the study. None of the participants had any hearing disorders or neurological disorders (based on self-report). All participants were recruited through an online survey company, Cross Marketing Inc. (2024), and were compensated for participation at a fixed rate determined by the company. The study was carried out in accordance with the Declaration of Helsinki and received approval from the Ethics Committee of The University of Tokyo (Approval No. UT-IST-RE-230601). Participants provided informed consent before starting the experiment, which they then completed online using a personal computer.

### 2.2 Stimuli

For auditory stimuli with varying structural complexity, we prepared sound sequences with pitch movement that followed varying global and local syntactic patterns. The created stimuli are publicly available in the Auditory_stimuli folder at https://osf.io/tpqnj/files/osfstorage.

#### 2.2.1 Sounds

To form sound sequences, we first created eight sounds. Each sound was a Shepard tone (Shepard, 1964), evenly spaced across one octave. The frequencies of the eight sounds were:

$$440*2^{\frac{i}{8}}\;\mathrm{Hz}\;(i=0,\ldots ,7)$$

All sounds had a duration of 400 ms, a constant loudness, and a sampling frequency of 44,100 Hz.

#### 2.2.2 Sound sequences

Using the created sounds, we formed sound sequences with pitch movements following varying global and local syntactic patterns. We adopted a stimuli design used in previous work, which provides an auditory parallel to Navon’s widely used visual local–global stimuli (Navon, 1977). The Navon figure is a visual stimulus in which a large, global shape (e.g., the letter “H”) is constructed from many smaller, local elements (e.g., the letter “S”). The design is used in various global and local auditory processing studies (Justus and List, 2005; List et al., 2007; Sanders and Poeppel, 2007; Ouimet et al., 2012; Bouvet et al., 2011).

**Figure 1:**
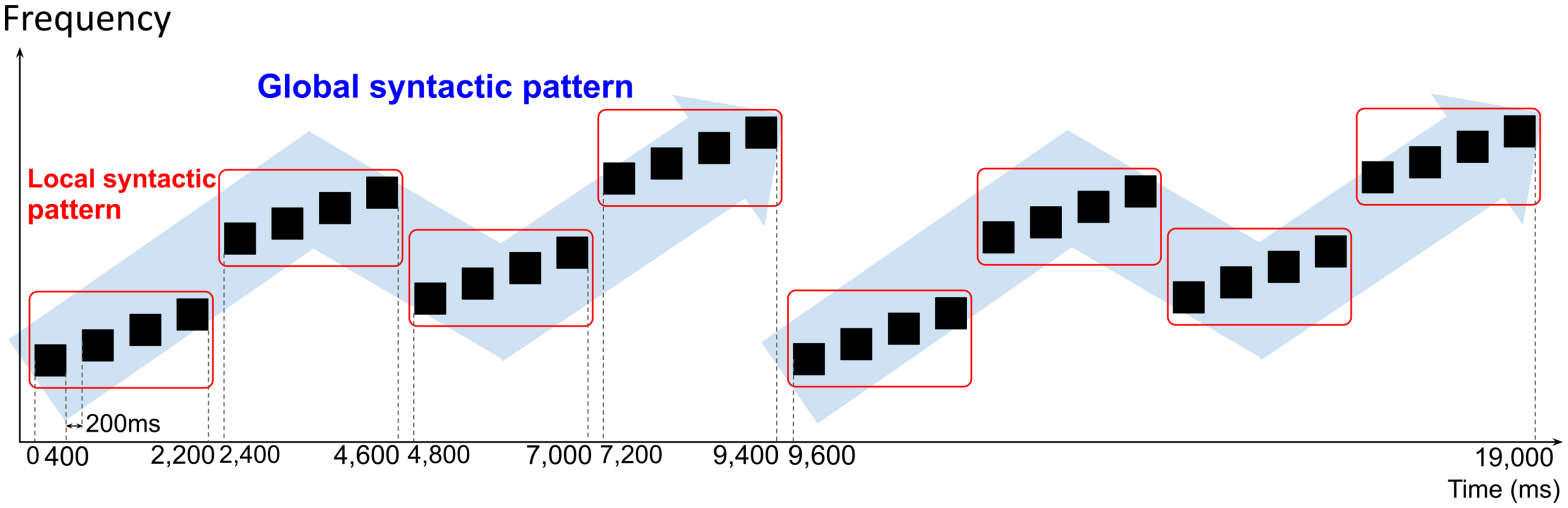
Example of sound sequence created for the auditory stimuli. The black boxes depict single tones in the sound sequence. Sound sequences consisted of two sets of four quadruplets. A quadruplet consisted of four single tones concatenated to follow a local syntactic pattern. Four quadruplets were concatenated to follow a global syntactic pattern. Each single tone had a duration of 400 ms and a 200-ms interval was placed between all single tones. The total duration of a sound sequence was 19 s.

Each sound sequence consisted of two sets of four quadruplets. A quadruplet consisted of four sounds that follow a local syntactic pattern. Four quadruplets were concatenated to follow a global syntactic pattern. The four-quadruplet sequence was repeated twice. A 200-ms interval was placed between all sounds in the sequence to make the sounds seem more independent of each other, and hence the local and global syntactic patterns less apparent. The total duration of a sound sequence was 19 s. Figure 1 depicts an example of a created sound sequence.

**Figure 2:**
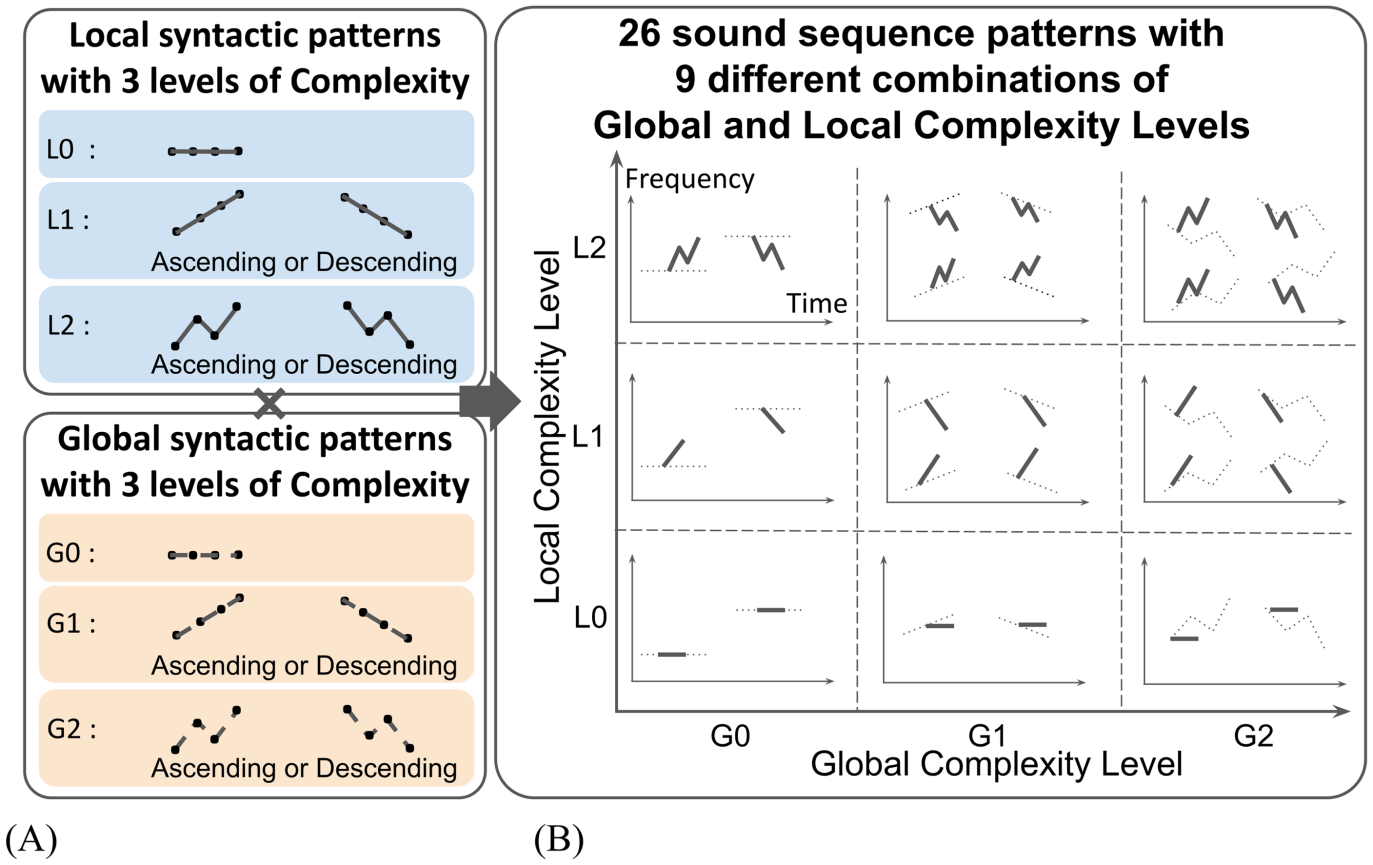
Sound sequences with systematically varied Local and Global Complexity. **(A)** Sound sequences were created by combining three levels of Local and Global Complexity in pitch movement (unchanged, linear, zigzag) with two variations in pitch direction (ascending, descending). **(B)** As a result, a total of 26 sound sequence patterns across nine distinct complexity categories were produced.

The local syntactic patterns followed three types of pitch movements that differed in complexity: static (unchanged), linear, and zigzag (Figure 2A). The static pattern was assigned a Complexity Level of L0, the linear pattern a Complexity Level of L1, and the zigzag pattern a Complexity Level of L2. Within the L1 and L2 patterns (linear and zigzag movement), ascending and descending variations were created. Similarly, global syntactic patterns followed the same three types of pitch movements: static (G0), linear (G1), and zigzag (G2). The same ascending/descending variations were created for the G1 and G2 patterns (Figure 2A).

Combining the local and global syntactic patterns, nine types of sound sequences patterns were produced: L0xG0, L0xG1, L0xG2, L1xG0, L1xG1, L1xG2, L2xG0, L2xG1, L2xG2. Ascending/descending variations resulted in two versions each for L0xG1, L0xG2, L1xG0, and L2xG0 patterns, and four versions each for L1xG1, L1xG2, L2xG1, and L2xG2 patterns. For the L0xG0 pattern, two variations using sounds with different pitches were created in order to reduce a preference for a specific sound. As a result, a total of 26 sound sequences patterns were created. A table of created sound sequence patterns is depicted in Figure 2B.

To minimize the possibility that differences in emotional experience across sequences were driven by pitch content rather than complexity level, the sounds used to construct each sound sequence pattern were selected so that sequences began with a range of different pitches as much as possible—avoiding repeated use of a single starting pitch—while still ensuring that the intended syntactic structure was preserved using the available eight sounds.

### 2.3 Measures

#### 2.3.1 Experienced emotion

Emotions experienced by listening to the sound sequences were quantified with the two-dimensional model of valence and arousal proposed by Russell (1980). Valence and arousal are widely recognized as fundamental dimensions of emotions and have been shown to account for the majority of observed variance in the emotional labeling of several types of experimental stimuli, including linguistic, pictorial, and musical (Gomez and Danuser, 2007; Coutinho and Cangelosi, 2011). Valence and arousal were each rated on a 9-point Likert scale, with valence ranging from 1 (felt negative emotion/unpleasantness) to 9 (felt positive emotion/pleasantness), and arousal from 1 (emotion was not felt at all) to 9 (emotion was felt very strongly).

#### 2.3.2 Bodily sensations

**Figure 3:**
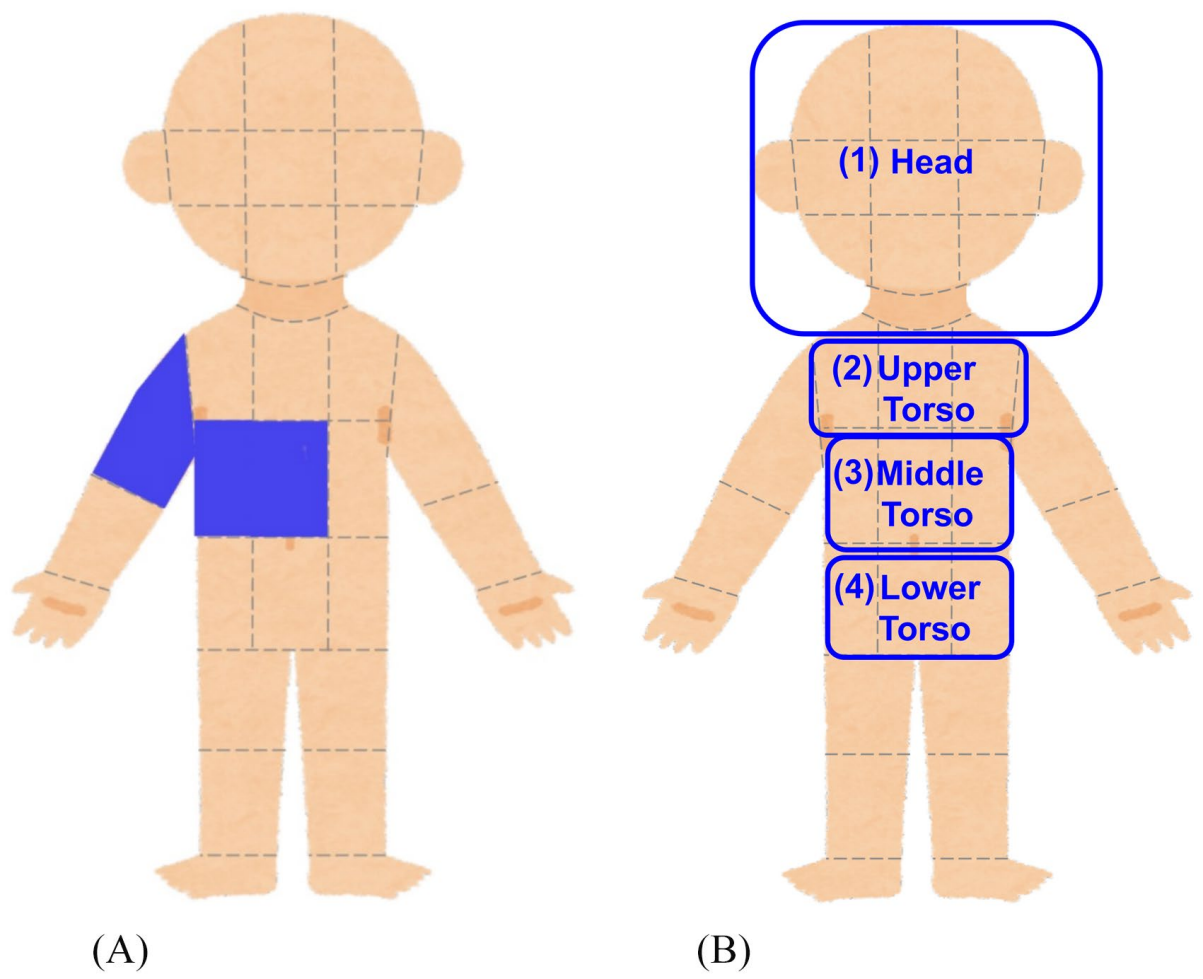
Bodily map and grouping of body sections. **(A)** Participants clicked on the body sections where they felt (or expected to feel) bodily sensations while listening to the presented sound sequences. **(B)** The body sections were grouped into broader body areas (Head, Upper Torso, Middle Torso, Lower Torso) which are suggested to be associated with emotional response to auditory stimuli.

Bodily sensations felt by listening to the sound sequences were measured by using an adaptation of the bodily map of emotion proposed by Nummenmaa et al. (2014). Participants were presented with a body image divided into 33 sections and were asked to select at least one section where they felt bodily sensation while they listened to the presented sound sequence (or where they would feel a bodily sensation if they continued to listen to) (Figure 3A).

#### 2.3.3 Listener features

As listener features, we obtained the participants’ sensory profile, autistic traits, intolerance of uncertainty, age, and gender.

The sensory profile was assessed using the Japanese version of the Adolescent-Adult Sensory Profile (AASP) (Brown et al., 2001). The AASP is a 60-item questionnaire designed to evaluate sensory processing across six sensory domains: taste/smell, movement, visual, touch, activity level, and hearing. It provides scores for four sensory processing patterns, each representing a combination of neurological threshold and behavioral response to sensory input: Low Registration (high threshold x passive behavior), Sensation Seeking (high threshold x counteractive behavior), Sensory Sensitivity (low threshold x passive behavior), and Sensation Avoiding (low threshold x counteractive behavior). Each sensory processing pattern score ranges from 15 to 75, with higher scores indicating a stronger tendency toward the corresponding sensory processing pattern. It is worth noting that the four sensory processing patterns are independent but not mutually exclusive of each other, i.e., individuals can exhibit the characteristics of multiple sensory processing patterns, although some patterns may seem contradictory (e.g., Sensory Sensitivity and Low Registration) (Ranford et al., 2020). We chose to use this measure because it captures both typical and atypical sensory processing patterns, accounting for listener characteristics such as age, but without focusing exclusively on any specific atypical condition or disorder to explain sensory processing patterns (Dean et al., 2022).

Autistic traits were assessed using the Japanese short form of the Autism Spectrum Quotient (AQ-J-10). The AQ-J-10 is a 10-item self-report questionnaire derived from Baron-Cohen et al.'s (2006) AQ questionnaire. It was developed by Kurita et al. (2005) for screening patients with high-functioning pervasive developmental disorder, now classified under autism spectrum disorder (American Psychiatric Association, 2013). The AQ-J-10 score ranges from 0 to 10, with higher scores indicating stronger autistic traits. Autistic traits have been associated with differences in how individuals process hierarchical information, particularly in how local and global levels are weighted and flexibly integrated. For example, Mottron et al. (2000) found that individuals with ASD showed enhanced local processing of musical features while maintaining intact global processing, suggesting a local bias rather than a global deficit. Similarly, Sapey-Triomphe et al. (2023) found that while higher-level (i.e., more global) predictions were intact in individuals with ASD, they were encoded more rigidly and with reduced adaptability to change.

Intolerance of uncertainty was assessed with the Japanese version of the Short Intolerance of Uncertainty Scale (SIUS), a 12-item questionnaire developed by Takebayashi et al. (2012) based on previous English versions (Carleton et al., 2007; Freeston et al., 1994). The SIUS score ranges from 12 to 60, with a higher score indicating greater intolerance of uncertainty. Uncertainty has been associated with the processing of hierarchical information. Previous findings suggest that learning hierarchical structures in music can reduce predictive uncertainty, particularly in relatively simple, low-entropy contexts (Hansen and Pearce, 2014). Under high uncertainty, the brain incorporates both local adjustment cues and global statistical regularities to form and update predictions (Kluger et al., 2020). The association of uncertainty with ASD has also been highlighted in findings showing that intolerance of uncertainty is significantly higher in children with ASD than in neurotypical peers and is positively correlated with core autism traits such as repetitive behaviors, social communication deficits, and emotion dysregulation (Vasa et al., 2018). Additionally, individuals with ASD have been found to show less flexible adjustment to prediction errors when processing expected and unexpected uncertainty, particularly under volatile conditions (Sapey-Triomphe et al., 2023).

#### 2.3.4 Other measures

In addition to the primary measures listed above, supplementary data were collected to assess participants’ emotional experiences and listener characteristics. However, these measures were not included in the present analyses for the reasons outlined below.

As supplementary indices of emotional experience, participants provided: (1) valence and arousal ratings for each single tone, assessed using the same 9-point Likert scales as those used for the sound sequences; (2) subjective ratings of the noisiness and complexity of each sound sequence, each assessed on a 4-point Likert scale; and (3) self-reported emotional categories associated with each sound sequence, selected from a list of 18 options (e.g., “happy,” “sad”), with up to three categories permitted per trial.

As an additional listener feature, participants reported their musical experience, quantified as the number of years spent receiving musical education or performing music outside the school curriculum. A brief description of the content of their musical experience was also obtained.

The collected emotion ratings for the single tones and the subjective ratings of noisiness, complexity, and emotion categories for the sound sequences were not included in the present analyses as they fall outside the scope of this study. Musical experience was also not included in the analyses due to the small number of participants with musical experience compared to those without.

The complete raw dataset, including these additional data, is publicly available in the Raw_data folder at https://osf.io/tpqnj/files/osfstorage.

### 2.4 Procedures

The experimental paradigm was created using the Gorilla Experiment Builder (Anwyl-Irvine et al., 2020), an online behavioral experiment builder. Participants accessed the experimental website using their personal computers from a location of their choice, were briefed on the details of the experiment in writing on the screen, consented to participation, completed screening questions, and started the experiment. The participants first completed the questionnaire on their listener features as mentioned above. Then they moved on to the stimuli rating task, where they were presented with the 8 single sounds and subsequently asked to provide their emotional response to each tone. Next, the 26 sound sequences were presented and participants were subsequently asked to provide their emotional and bodily response to each sound sequence. The single sounds and sound sequences were presented in randomized order for each participant.

### 2.5 Statistical analyses

Statistical analyses were conducted using Jamovi Version 2.3 (Şahin and Aybek, 2019; The Jamovi Project, 2024). Participants with missing responses were omitted prior to the analyses.

For each participant, the valence and arousal ratings were averaged across sound sequences with the same Local and Global Complexity Levels, producing nine averaged values for each rating (valence and arousal ratings for sound sequences with Complexity Level of L0xG0, L0xG1, L0xG2, L1xG0, L1xG1, L1xG2, L2xG0, L2xG1, L2xG2).

We assessed the normality of valence and arousal ratings using the Shapiro–Wilk test, skewness and kurtosis values, as well as visual inspection of histograms and Q-Q plots. Based on these results, we decided whether to use either a parametric or non-parametric repeated-measures analysis of variance (ANOVA) to evaluate the effects of various factors on the valence and arousal ratings, respectively.

First, repeated-measures ANOVAs were conducted to examine the effects of structure features, listener features, and bodily sensations on valence ratings. In all ANOVAs, the dependent variable was the valence score, with two within-subject factors and two between-subject factors. The within-subject factors were: (1) the Local Complexity Level (L0, L1, L2) of the sound sequences, and (2) the Global Complexity Level (G0, G1, G2) of the sound sequences. The between-subject factors varied across ANOVAs and consisted of (1) the score of one AASP sensory processing pattern and (2) the presence or absence of bodily sensation in a specific body area. The specific AASP sensory processing patterns and bodily sensation areas used in the analysis are shown in Table 1. The thresholds for dividing participants into high and low groups for each sensory processing pattern were set to ensure an equal split, with half of the participants in each group. The bodily sensation factors correspond to the following body areas: (1) Head, (2) Upper Torso, (3) Middle Torso, and (4) Lower Torso, as illustrated in Figure 3B. Participants who reported a bodily sensation in a specific body area for at least one sound sequence were classified into the group *with* bodily sensation in that body area. For example, a participant who reported a bodily sensation in the Head area for three sound sequences was categorized as a member of the *with bodily sensation in the Head* group. These four body areas were selected based on prior research suggesting connections between these areas and emotional responses (Daikoku et al., 2024; Nummenmaa et al., 2014; Putkinen et al., 2024).

**Table 1:** Between-subject factors in ANOVA.

Score of AASP sensory processing pattern	Presence of bodily sensation in body area
Low registration (high vs. low)	Head (with vs. without)
Sensation seeking (high vs. low)	Upper torso (with vs. without)
Sensory sensitivity (high vs. low)	Middle torso (with vs. without)
Sensation avoiding (high vs. low)	Lower torso (with vs. without)

Secondly, repeated-measures ANOVAs were conducted to evaluate the effects of structure features, listener features, and bodily sensations on arousal ratings. The dependent variables were arousal ratings. The within- and between-subject factors were the same as those used in the ANOVAs for valence ratings.

For all ANOVAs, the significance level was set at 5%. We used post-hoc contrasts to follow up statistically significant interactions (and/or main effects with more than 2 levels), The *p*-values of post-hoc tests were adjusted based on the false discovery rate. Effect sizes were estimated using partial eta squared(*η_p_*^2^).

In addition, we conducted independent samples t-tests to examine the differences in AQ and SIUS scores in high and low score groups of each AASP sensory processing patterns. The significance level for the t-tests were set at 5%.

## 3 Results

A total of 579 participants (female = 283, male = 294, other = 2; mean age = 35.88 years, SD = 8.86) were included in the statistical analysis after excluding those with missing responses. The complete raw dataset for participants with complete responses, along with all analysis results, is publicly available at https://osf.io/tpqnj/.

### 3.1 ANOVA results

We first examined the distribution of the valence and arousal ratings. The Shapiro–Wilk test showed significant deviations from normality (*p <* 0.001). However, given the large sample size (*>* 500) and a W statistic close to 1, these deviations were not considered problematic. Additionally, skewness and kurtosis fell within the acceptable range for normality (between −2 and +2) (George and Mallery, 2010). Considering these factors along with visual inspection of histograms and Q-Q plots, we assumed that the data could be sufficiently approximated by a normal distribution (histograms and Q-Q plots can be found in the Analysis_results folder at https://osf.io/tpqnj/files/osfstorage). Based on this assumption, we conducted the parametric repeated-measures ANOVA on the dataset.

#### 3.1.1 Valence

The significant effects identified across all ANOVA models are summarized in Supplementary Table 3. Full statistical descriptors for each effect in the table are available in the files located at Analysis_results/ANOVA/Valence in the OSF repository: https://osf.io/tpqnj/files/osfstorage. In this subsection, we focus mainly on the ANOVA that included Sensory Sensitivity score and Upper Torso area (with vs. without Upper Torso sensation) as between-subject factors, as this model yielded the greatest number of significant and interpretable effects.

The ANOVA model with Sensory Sensitivity score and Upper Torso area as between-subject factors revealed a series of significant effects, beginning with a main effect of Local Complexity Level that was significant with *F*(2, 1150) = 5.04 (*p* = 0.007, *η_p_*^2^ = 0.009). Post-hoc tests revealed that valence ratings of sound sequences with Local Complexity Level of L1 (medium) were significantly higher than those of L0 (low) and L2 (high) (L0: *p* = 0.035, L2: *p* = 0.003) (Supplementary Figure 1). The main effect of Local Complexity Level was significant in all other models as well (i.e., in all combinations of sensory processing pattern scores and bodily sensation areas used as between-subject factors), and the L1 valence was also significantly higher than L2 valence in all models (*p <* 0.04).

The main effect of Global Complexity Level was also significant with *F*(2,1150) = 72.40 (*p* < 0.001, *η_p_*^2^ = 0.112). Post-hoc tests revealed that the valence ratings of sound sequences with Global Complexity Level of G0 (low) were lower than those of both G1 (medium) and G2 (high) (*p* < 0.002) (Supplementary Figure 2). The main effect of Global Complexity Level was significant in all other models as well, and valence ratings of G0 sequences were also lower than those of both G1 and G2 in all models (*p <* 0.002).

**Figure 4:**
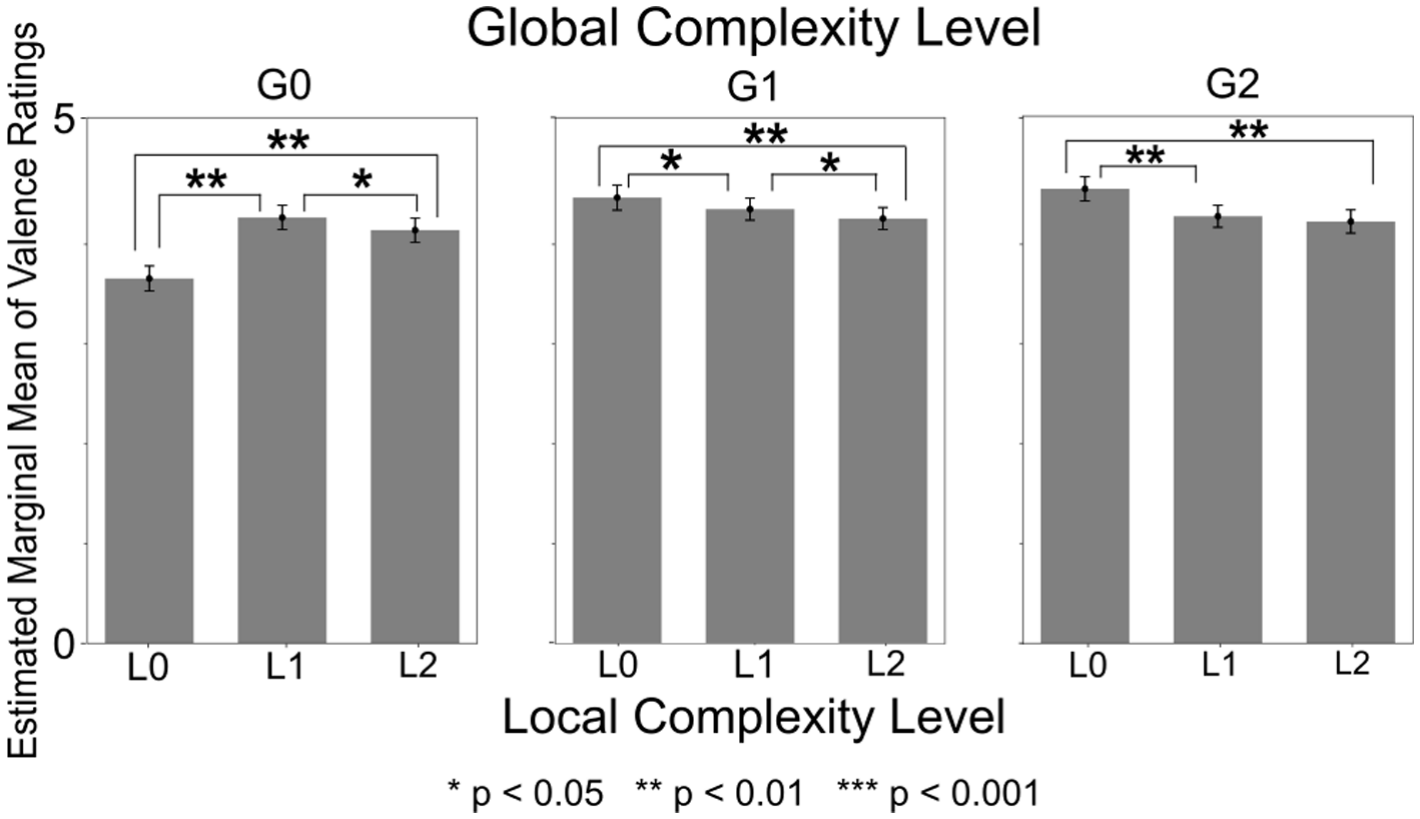
Valence ratings by local complexity level x global complexity level. Valence ratings were significantly higher for sound sequences with high Local and low Global Complexity (L1xG0, L2xG0) compared to sequences with low Local and low Global Complexity (L0xG0). In contrast, valence ratings were higher for sequences with low Local and medium or high Global Complexity (L0xG1, L0xG2) compared to sequences with high Local and medium or high Global Complexity (L1xG1, L2xG1, L1xG2, L2xG2).

The within-subject interaction of Local Complexity Level and Global Complexity Level was significant with *F*(4,2300) = 57.01 (*p* < 0.001, *η_p_*^2^ = 0.090) (Figure 4). Post-hoc tests revealed that, among sound sequences with Global Complexity Level of G0 (low), the valence ratings were lowest for sequences with Local Complexity Level of L0 (low). Among sound sequences with Global Complexity Level of G1 (medium), the valence ratings were the lowest for sequences with Local Complexity Level of L2 (high). Among sound sequences with Global Complexity Level of G2 (high), the valence ratings were the highest for sequences with Local Complexity Level of L0 (low). Additionally, the L0xG0 sound sequences (lowest local and global complexity) received the lowest overall valence ratings. The within-subject interaction of Local Complexity Level and Global Complexity Level was significant in all other models as well, and the post-hoc test results were also significant in all models.

The significant main effects for both Sensory Sensitivity score [*F*(1,575) = 4.56, *p* = 0.033, *η_p_*^2^ = 0.008] and Upper Torso area [*F*(1,575) = 11.27, *p <* 0.001, *η_p_*^2^ = 0.019]. However, the interaction between Sensory Sensitivity score and Upper Torso area was insignificant (*p* = 0.852).

**Figure 5:**
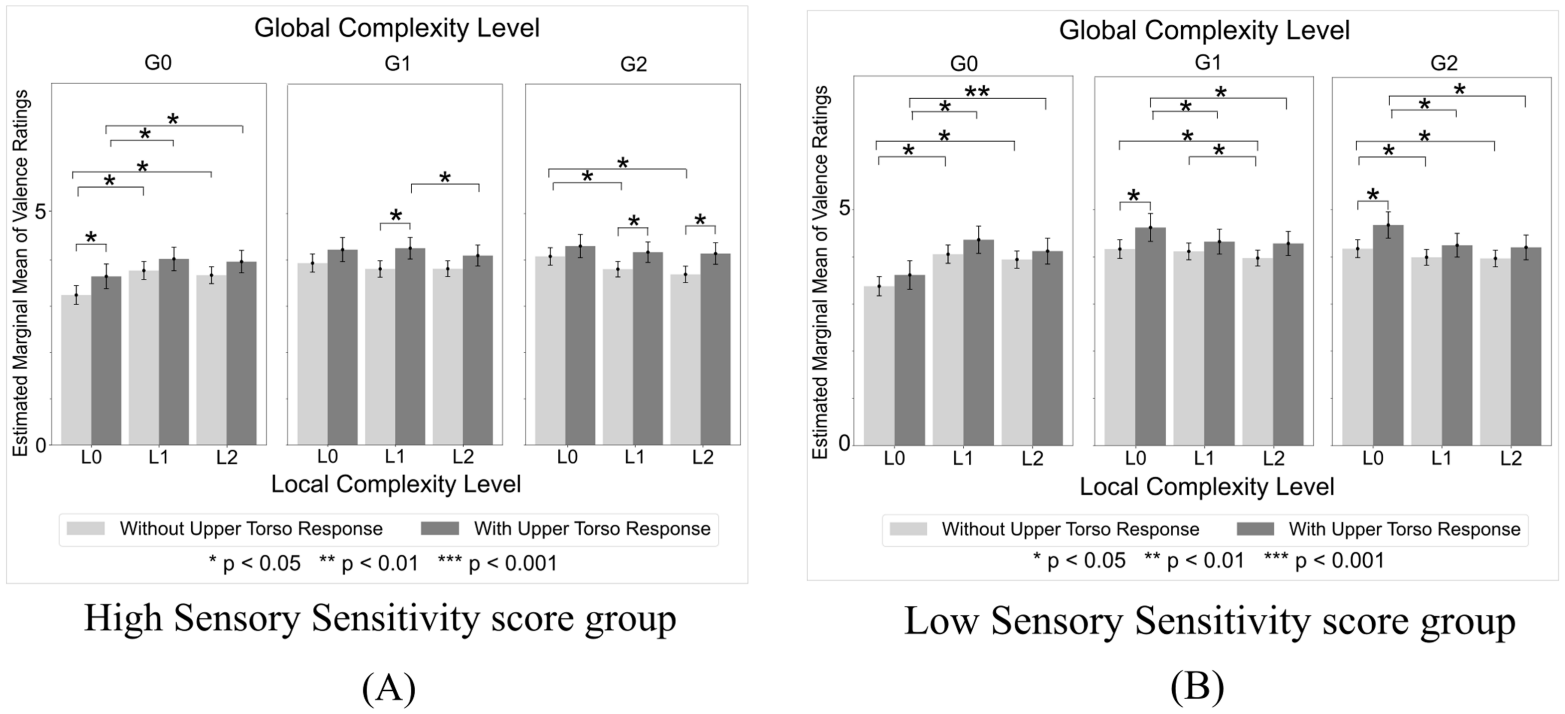
Valence ratings by local complexity level x global complexity level in high and low sensory sensitivity score groups. **(A)** In the high Sensory Sensitivity score group, participants who experienced Upper Torso sensations showed significantly higher valence ratings for sequences with higher combinations of Local and Global Complexity levels compared to those who did not experience Upper Torso sensations. **(B)** By contrast, in the low Sensory Sensitivity score group, participants who experienced Upper Torso sensations showed significantly higher valence ratings for sequences with lower Local and higher Global Complexity levels.

The Local Complexity Level x Global Complexity Level x Sensory Sensitivity score x Upper Torso area interaction was significant [*F*(4,2300) = 2.48, *p* = 0.042, *η_p_*^2^ = 0.004]. Post-hoc comparisons revealed that valence ratings were higher in the low Sensory Sensitivity score group than in the high Sensory Sensitivity score group, and higher in participants who reported Upper Torso bodily sensations compared to those who did not. Among participants with high Sensory Sensitivity scores, those who experienced Upper Torso sensations showed significantly higher valence ratings for sequences with higher combinations of Local and Global Complexity Levels (L1xG1, *p* = 0.014; L1xG2, *p* = 0.028; L2xG2, *p* = 0.010) compared to those who did not experience Upper Torso sensation (Figure 5A). Furthermore, the decline in valence ratings for G2 sequences as Local Complexity Level increased was significantly smaller in participants with Upper Torso sensations than in those without. Specifically, among participants *without* Upper Torso sensation, the mean valence difference between L0xG2 and L1xG2 sequences was 0.28 (*p* = 0.011), and between L0xG2 and L2xG2 sequences was 0.39 (*p* = 0.011). In contrast, among participants *with* Upper Torso sensations, no significant differences in valence were observed between L0xG2 and L1xG2 sequences (*p* = 0.208) or L0xG2 and L2xG2 sequences (*p* = 0.142). Conversely, among participants with low Sensory Sensitivity scores, those who experienced Upper Torso sensations showed significantly higher valence ratings for sequences with lower Local and higher Global Complexity Levels (L0xG1, *p* = 0.031; L0xG2, *p* = 0.015) compared to those who did not experience Upper Torso sensation (Figure 5B). Furthermore, the decline in valence ratings of G1 and G2 sequences as Local Complexity Level increased was significantly larger in participants with Upper Torso sensation compared to those without. Specifically, in participants *with* Upper Torso sensations, the mean valence difference between L0xG1 and L2xG1 sequences was 0.34 (*p* = 0.015), and between L0xG2 and L2xG2 sequences was 0.47 (*p =* 0.011). In contrast, among participants *without* Upper Torso sensations, the mean valence difference between L0xG1 and L2xG1 sequences was 0.19 (*p* = 0.037), and between L0xG2 and L2xG2 sequences was 0.21 (*p* = 0.015).

Similarly, ANOVA with Sensory Sensitivity score and Middle Torso area as between-subject factors revealed significant main effects for both Sensory Sensitivity score [*F*(1,575) = 5.73, *p* = 0.017, *η_p_*^2^ = 0.01] and Middle Torso area [*F*(1,575) = 7.11, *p* = 0.008, *η_p_*^2^ = 0.012], and a non-significant Sensory Sensitivity x Middle Torso interaction (*p* = 0.652). The Local Complexity Level x Global Complexity Level x Sensory Sensitivity score x Middle Torso area interaction was also significant [*F*(4,2300) = 2.42, *p* = 0.047, *η_p_*^2^ = 0.004]. Post-hoc comparisons revealed that valence ratings were higher in the low Sensory Sensitivity score group compared to the high Sensory Sensitivity score group. Post-hoc comparisons also revealed that valence ratings were higher in participants with Middle Torso bodily sensation compared to participants without. Among participants with high Sensory Sensitivity scores, those who experienced sensations in the Middle Torso showed higher valence scores than those who did not, although the difference was not statistically significant.

Other significant effects and interactions observed were as follows: ANOVA with Sensation Avoiding score and Head area as between-subject factors revealed significant main effects for Sensation Avoiding scores [*F*(1,575) = 5.64, *p* = 0.018, *η_p_*^2^ = 0.01] and Head area [*F*(1,575) = 4.19, *p* = 0.041, *η_p_*^2^ = 0.007], with low Sensation Avoiding scores and presence of Head sensations yielding higher valence scores. ANOVA with Sensation Avoiding score and Middle Torso area as between-subject factors revealed significant main effects for Sensation Avoiding score [*F*(1,575) = 6.41, *p* = 0.012, *η_p_*^2^ = 0.011] and Middle Torso area [*F*(1,575) = 7.01, *p* = 0.008, *η_p_*^2^ = 0.012], with low Sensation Avoiding scores and presence of Middle Torso sensations yielding higher valence scores. ANOVA with Sensation Seeking scores and Upper Torso area as between-subject factors revealed significant main effects for Sensation Seeking score [*F*(1,575) = 6.83, *p* = 0.009, *η_p_*^2^ = 0.012] and Upper Torso area [*F*(1,575) = 8.26, *p* = 0.004, *η_p_*^2^ = 0.014], with high Sensation Seeking scores and presence of Upper Torso sensations yielding higher valence scores. The sensory processing pattern x bodily sensation area interactions were insignificant in all above combinations.

#### 3.1.2 Arousal

The significant effects identified across all ANOVA models are summarized in Supplementary Table 4. Full statistical descriptors for each effect in the table are available in the files located at Analysis_results/ANOVA/Arousal in the OSF repository: https://osf.io/tpqnj/files/osfstorage. In this subsection, we focus mainly on results obtained from the ANOVA that included Sensation Seeking score and Upper Torso area as between-subject factors, as this model yielded the greatest number of significant and interpretable effects.

**Figure 6:**
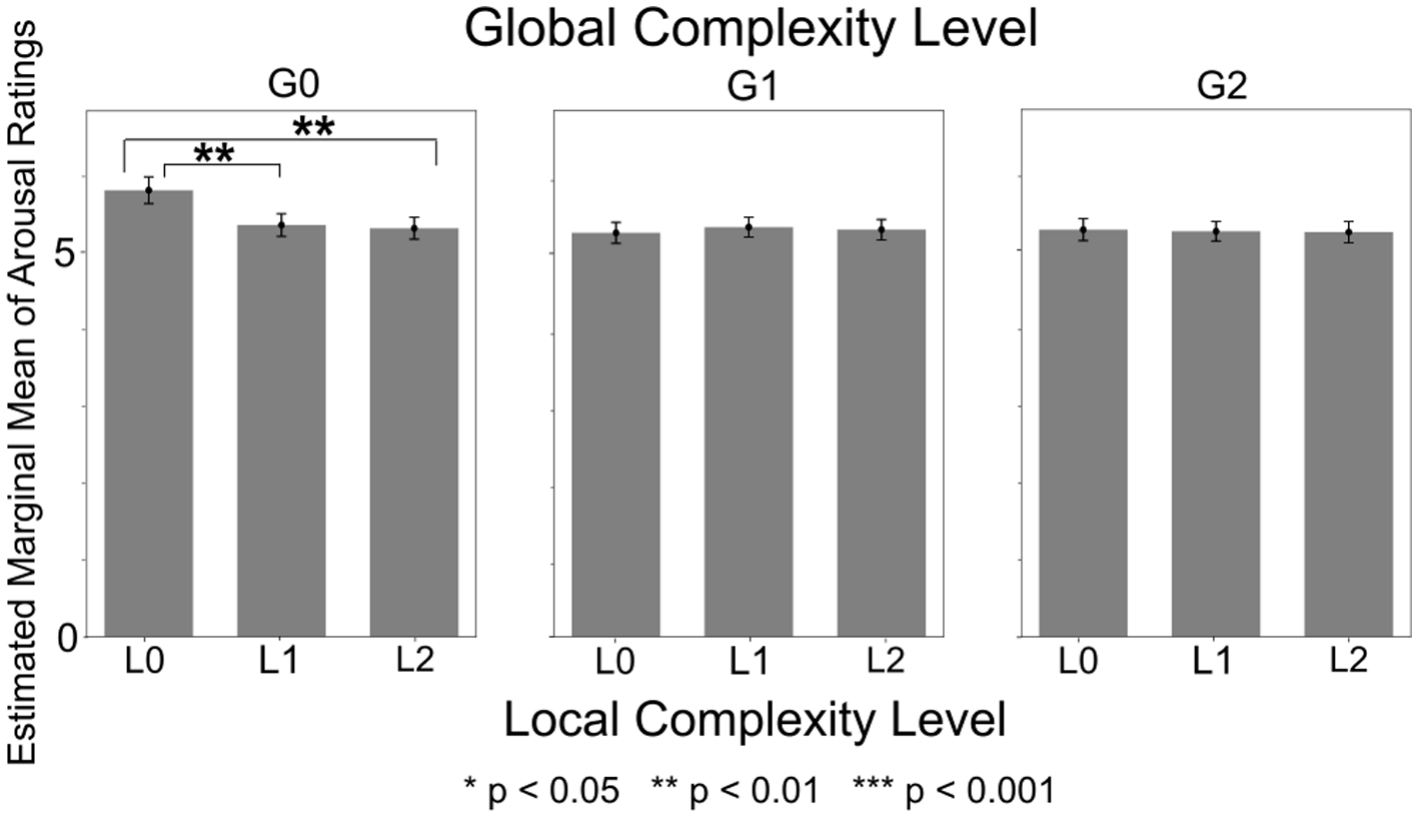
Arousal ratings by local complexity level x global complexity level. The sound sequences with lowest Local and lowest Global Complexity (L0xG0) elicited significantly higher arousal compared to sequences with other Lower x Global Complexity combinations.

The ANOVA model with Sensation Seeking score and Upper Torso area as between-subject factors revealed significant main effects of Local Complexity Level [*F*(2,1150) = 17.36, *p* < 0.001, *η_p_*^2^ = 0.029]. Post-hoc tests revealed that arousal was highest for L0 sound sequences (lowest Local Complexity Level) (*p* = 0.002) (Supplementary Figure 3). The main effect of Global Complexity Level was also significant [*F*(2,1150) = 31.55, *p* < 0.001, *η_p_*^2^ = 0.052], with post-hoc tests revealing that arousal was highest for G0 sound sequences (lowest Global Complexity Level) (*p* = 0.002) (Supplementary Figure 4). The within-subject interaction of Local Complexity Level and Global Complexity Level was also significant [*F*(2,1150) = 22.59, *p* < 0.001, *η_p_*^2^ = 0.038]. Post-hoc tests revealed that L0xG0 sound sequences (lowest Local and Global Complexity Levels) elicited significantly higher arousal compared to sequences with other Local x Global Complexity combinations, as shown in Figure 6 (*p* = 0.002). The main effects of Local Complexity Level and Global Complexity Level, as well as their interaction, were significant in all ANOVA models (i.e., all combinations of sensory processing pattern scores and bodily sensation areas used as between-subject factors).

**Figure 7:**
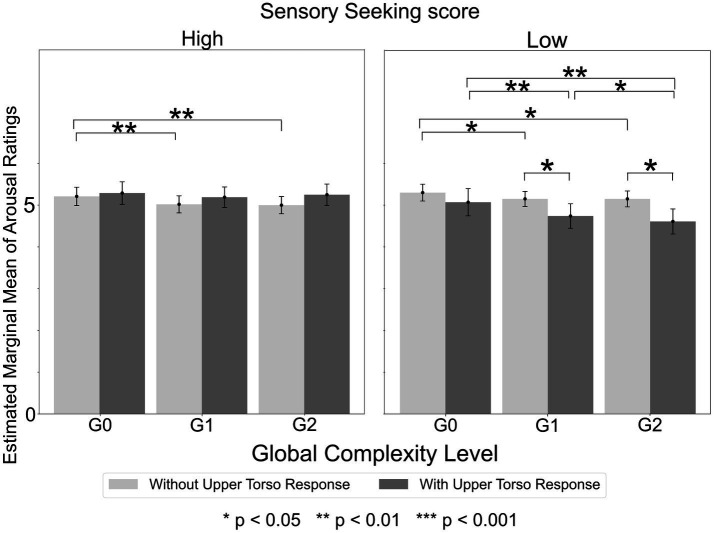
Arousal ratings by global complexity level in high and low sensation seeking score groups. In the low Sensation Seeking score group, participants who experienced Upper Torso sensation showed a significantly greater decrease in arousal ratings as Global Complexity Level increased, compared to those who did not experience Upper Torso sensations. However, no significant differences were found within the high Sensation Seeking score group.

Furthermore, the ANOVA with Sensation Seeking score and Upper Torso area as between-subject factors revealed a significant Global Complexity Level x Sensory Sensitivity score x Upper Torso area interaction [*F*(2,1150) = 7.91, *p* < 0.001, *η_p_*^2^ = 0.014]. Post-hoc tests revealed that, within the low Sensation Seeking group, participants who experienced Upper Torso sensation exhibited a significantly greater decrease in arousal ratings as Global Complexity Level increased, compared to participants who did not experience Upper Torso sensations (Figure 7). Specifically, among participants with Upper Torso sensations, mean arousal ratings for G1 and G2 sequences were 0.36 and 0.46 lower than that for G0 sequences (*p* = 0.009). In contrast, among participants without Upper Torso sensations, the corresponding differences were only 0.15 and 0.15 (*p* = 0.010). Consequently, the arousal ratings for G1 and G2 sequences were significantly lower in participants with Upper Torso sensations compared to those without (G1: *p* = 0.041, G2: *p* = 0.01). However, no such significant mean differences were found within the high Sensation Seeking group.

In addition to the above, some notable results were observed in other ANOVA models. A significant main effect of Sensation Avoiding scores was found in all ANOVAs with the Sensation Avoiding score as the between-subject factor. Regardless of the body area selected as the between-subject factor, the main effect of Sensation Avoiding score was significant, with the descriptively largest *F*-statistic found for the Sensation Avoiding score and Middle Torso area combination [*F*(1,575) = 6.12, *p* = 0.014*, η_p_*^2^ = 0.011]. Post-hoc comparisons showed that arousal ratings were higher in the high Sensation Avoiding score group compared to the low Sensation Avoiding score group.

Other notable results included the interactions of Global Complexity Level x Sensation Seeking score x Middle Torso area, Global Complexity Level x Sensory Sensitivity score x Middle Torso area, and Global Complexity Level x Low Registration score x Middle Torso, in which participants with low sensory processing pattern scores who experienced bodily sensations consistently showed a greater decrease in arousal scores as Global Complexity Level increased from G0 to either G1 or G2.

### 3.2 *T*-test results

Welch’s independent t-tests were conducted due to significant results of Levene’s test for equality of variances for some AQ and SIUS scores. Participants with high Sensory Sensitivity scores had significantly higher AQ scores (*M =* 4.63, SD *=* 2.31) than those with low Sensory Sensitivity scores (*M =* 3.15, SD *=* 1.84), *t* (566) *=* 8.52, *p* < 0.001, *Cohen’s d =* 0.71. Similarly, participants with high Sensory Sensitivity scores had significantly higher SIUS scores (*M* = 36.99, SD = 7.45) than those with low Sensory Sensitivity scores (*M* = 31.47, SD = 7.35), *t* (574) = 8.97, *p <* 0.001, *Cohen’s d* = 0.75.

A similar pattern was observed when participants were grouped by Sensation Avoiding and Low Registration scores, with both showing significant differences (*p* < 0.001). However, no significant differences were found between AQ or SIUS scores for participants with high versus low Sensation Seeking scores (AQ: *p* = 0.45, SIUS: *p* = 0.93). These results are depicted in Supplementary Figures 5–8.

## 4 Discussion

The current study aimed to elucidate how emotional experiences are influenced by the global and local syntactic structure of auditory sequences in corroboration with listeners’ sensory processing patterns and auditory-induced bodily sensations. Participants were presented with sound sequences which had various levels of local and global complexity in their syntactic patterns. Emotions induced by the sound sequences were quantified by valence and arousal ratings.

Experimental results showed that emotional experience induced by a sound sequence is influenced by its hierarchical structure. Valence was higher for high Local x low Global Complexity and low Local x high Global Complexity structures. This result indicates that listeners preferred sound sequences with hierarchical structures that are in the Goldilocks zone, in other words, “not too simple, not too complex.”

Meanwhile, arousal was highest for lowest Local x lowest Global Complexity structure, which received the lowest valence rating across all structures. Sound sequences of this structure were a repetition of same sound, sounding like the beeping of an alarm. This result suggests that such sequences were highly alarming but unpleasant to the listeners.

Results showed that listener’s sensory processing patterns influence emotional experience. Participants with higher Sensory Sensitivity and Sensation Avoiding scores reported lower valence across all sound sequences compared to those with lower scores. Moreover, participants with higher Sensation Avoiding scores reported higher arousal. Given that both Sensory Sensitivity and Sensation Avoiding are sensory processing patterns classified as “low neurological threshold” patterns in the AASP framework—patterns in which individuals report heightened awareness of even low-intensity stimuli—these results suggest that individuals with such heightened awareness are more likely to experience negative feelings when exposed to auditory stimuli, and that these feelings are more strongly activated in those who exhibit a tendency to avoid sensory stimuli. On the other hand, participants with higher Sensation Seeking scores reported higher valence compared to those with lower scores. This suggests that participants who crave sensory stimulation tend to feel more pleasure when they are exposed to auditory stimuli.

Further results showed that bodily sensation, especially in the Upper Torso area, also has influence on emotional experience. In terms of valence, within participants with high Sensory Sensitivity, those who experienced Upper Torso sensation felt (1) higher valence in sound sequences with *higher* Local and higher Global Complexity and (2) significantly *decreased* valence difference between low Local x high Global and high Local x high Global Complexity sequences, whereas those who did not experience Upper Torso sensation felt (1) higher valence in sound sequences with *lower* Local and higher Global Complexity and (2) significantly *increased* valence difference between low Local x high Global and high Local x high Global Complexity sequences. In terms of arousal, within the low Sensation Seeking group, participants who experienced Upper Torso sensation exhibited a significantly greater decrease in arousal as the sound sequences’ Global Complexity Level increased. These results indicate that an interplay of the sound sequence’s hierarchical structure (Local and Global), listener’s sensory processing pattern (Sensory Sensitivity and Sensation Seeking), and presence of Upper Torso sensation leads to different outcomes in emotional experience (valence or arousal). The results also suggest that the Upper Torso area may be a key body area in auditory-induced emotional experience.

The results also suggest that the AASP sensory processing patterns overlap with autistic traits and intolerance of uncertainty, both of which have been linked to hierarchical structures, as mentioned earlier. Furthermore, the results indicate that the AASP sensory processing patterns capture Sensation Seeking traits, which were not reflected in the AQ scores but are known to be frequently observed in individuals with ASD (MacLennan et al., 2022). Therefore, the AASP sensory processing patterns can be considered to adequately capture listener features that are closely related to hierarchical syntactic structures.

The current study supports the previously proposed studies which suggest that emotional experiences are triggered by a combination of factors, including stimulus features and listener characteristics (Scherer and Zentner, 2001; Juslin and Västfjäll, 2008). The current study extends such studies by elucidating how hierarchical syntactic structure (global and local syntactic patterns) influence emotional experience. Specifically, we showed that a “not too simple, not too complex” structure is most preferred, as indicated by higher valence ratings. This is in accordance with the widely accepted inverted-U shape relationship between pleasure and stimulus complexity (Berlyne, 1971). The inverted-U shape relationship in audition was found in studies such as the one by Cheung et al. (2019), whose findings indicated that chords with low uncertainty and high surprise or with high uncertainty and low surprise were most pleasurable. Reber et al. (2004) explained the inverted-U preference through processing fluency, proposing that ease of processing leads to positive affect. They suggested that structures initially perceived as complex but ultimately easy to process evoke particularly strong pleasure. Applying this concept, our findings can be interpreted as follows: In high Local Complexity x low Global Complexity structures, high Local Complexity creates an impression of difficulty, while low Global Complexity facilitates actual processing. Conversely, in low Local Complexity x high Global Complexity structures, low Local Complexity makes the structure simpler, but high Global Complexity introduces processing difficulty.

The current study also adds to the previous auditory-emotion studies by clarifying listener features that have stronger relationships with auditory-induced emotions. Specifically, our study suggests that high Sensory Sensitivity reduces positive emotions towards auditory stimuli, which is in line with studies which indicate that individuals with high sensory sensitivity tend to be overwhelmed by environmental stimuli (Harrold et al., 2024).

Further, our study supports previously proposed connections between bodily sensation and auditory-induced emotion (James, 1884; Scherer and Zentner, 2001). Our study confirms the importance of the Upper Torso area, which covers the heart region. Moreover, and most significantly, our study suggests that the link between bodily sensation and emotional experience depends on a complex interplay with the audio’s hierarchical structure and listener’s sensory processing pattern. Specifically, our findings showed that the presence of Upper Torso sensation alters valence, but the effect differs by the Local and Global complexity of the audio structure and how strong the listener’s Sensory Sensitivity or Sensation Seeking trait is. Previous studies have demonstrated the influence of bodily sensations on emotion, particularly sensation in body parts included in the Upper Torso area, but their findings vary depending on other factors of emotion induction. For instance, Daikoku et al. (2024) found that heart-related sensations induced by an interplay of musical uncertainty and prediction error in musical chords were positively correlated with positive valence, whereas Putkinen et al. (2024) demonstrated the close link between chest areas and negative emotions with the auditory stimuli’s acoustic features coming into play. Taken together, these findings highlight that bodily sensations alone are not deterministic; instead, their emotional impact is shaped by a complex interplay with the auditory stimuli’s structure feature and listener feature. This underscores the need for an integrative approach in understanding the role of bodily sensations in emotion.

Although our study suggests a connection between the Upper Torso body area and auditory-induced emotions, the causal relationship remains unclear. Beyond the reported bodily sensations, it is not clear whether physiological responses in the reported areas were present while listening to the sounds. This could be addressed in future experiments where we record both subjective emotion ratings and physiological bodily changes induced by auditory sequences.

While we did observe various significant effects, one concern could be the multiple ANOVAs conducted in the analysis. This analytical approach was chosen based on the nature of our variables and research aims. Specifically, (1) the AASP sensory processing patterns are independent but not mutually exclusive—individuals who score high in one pattern (e.g., Sensory Sensitivity) may also score high in another (e.g., Low Registration); (2) bodily sensations reported across different areas are likewise not mutually exclusive—participants often reported sensations in more than one region (e.g., Head and Upper Torso); and (3) our primary interest was in examining the interaction between each sensory processing pattern and each bodily region, rather than exploring interactions among multiple patterns or regions simultaneously. To maintain interpretability and avoid conflating overlapping effects, we conducted separate ANOVAs for each combination. While the current study may be considered exploratory, these analyses provide initial insights that can inform more focused, hypothesis-driven investigations in future research.

Another concern could be that the effect sizes of the factors tested in the ANOVA of our current study were relatively small. This may be due to the influence of other well-established acoustic features, such as pitch and loudness, which are already known to significantly affect emotions but were not included in the current study. To accurately judge the impact of our current factors, one would need to analyze and compare the effect sizes of our current factors and such well-established factors together. Conducting a study that systematically incorporates factors identified as relevant in previous separate studies should be included in future works to provide a more comprehensive analysis of each factor’s individual impact.

Our study was one of the first to explore the association between hierarchical structure and emotion in audition. While we were able to show how the global and local syntactic patterns are related to emotional experience, higher-level structures created by incorporating patterns, such as the nesting of ascending/descending pitch movement at certain locations of the sound sequence, are yet to be explored. Such higher-level hierarchical structures are suggested to evoke tension and relaxation (Jackendoff and Lerdahl, 2006). Investigating emotional changes due to such higher-order hierarchical structures are expected to reveal further important findings.

In conclusion, the present study demonstrated that a complex interplay of audio structure feature, listener feature, and bodily sensations influence emotional experience induced by auditory stimuli. Findings provide empirical support for theories claiming multifactor triggers of auditory-induced emotion and connection between bodily sensation and emotion. Potential directions of future research include measuring physiological signals to investigate the causal relationship between bodily sensation and emotion and integrating all previously identified modulators into one study to comprehensively investigate the relevant emotion triggers.

## Data Availability

The datasets presented in this study can be found in online repositories. The names of the repository/repositories and accession number(s) can be found in the article/Supplementary material. Complete raw dataset and analysis results for this study can be found at https://osf.io/tpqnj/.
