# Supplementary material for: Emotional responses to auditory hierarchical structures is shaped by bodily sensations and listeners’ sensory traits
Source: Front Psychol. 2025 Jul 8;16:1599430. doi: 10.3389/fpsyg.2025.1599430 (PMC12279784; doi:10.3389/fpsyg.2025.1599430)
Supplement: Supplementary file 1 [file Table_1.docx]

Supplementary Material

# Supplementary Tables

**Supplementary Table 1. Descriptives of Valence Ratings**

1. Shapiro-Wilk test results of Valence Ratings

| **Local x Global complexity** | **Shapiro-Wilk W** | **Shapiro-Wilk p** |
| --- | --- | --- |
| L0xG0 | 0.964 | <.001 |
| L0xG1 | 0.965 | <.001 |
| L0xG2 | 0.969 | <.001 |
| L1xG0 | 0.973 | <.001 |
| L1xG1 | 0.977 | <.001 |
| L1xG2 | 0.973 | <.001 |
| L2xG0 | 0.975 | <.001 |
| L2xG1 | 0.979 | <.001 |
| L2xG2 | 0.979 | <.001 |

1. Skewness and kurtosis of Valence Ratings

| **Local x Global complexity** | **Skewness** | **Std. error skewness** | **Kurtosis** | **Std. error kurtosis** |
| --- | --- | --- | --- | --- |
| L0xG0 | 0.00824 | 0.102 | -0.586 | 0.203 |
| L0xG1 | -0.373 | 0.102 | -0.196 | 0.203 |
| L0xG2 | -0.312 | 0.102 | 0.272 | 0.203 |
| L1xG0 | -0.21 | 0.102 | -0.0431 | 0.203 |
| L1xG1 | -0.362 | 0.102 | 0.0757 | 0.203 |
| L1xG2 | -0.475 | 0.102 | -0.0205 | 0.203 |
| L2xG0 | -0.13 | 0.102 | -0.0615 | 0.203 |
| L2xG1 | -0.379 | 0.102 | 0.0279 | 0.203 |
| L2xG2 | -0.345 | 0.102 | -0.0643 | 0.203 |

1. Mean and Median of Valence Ratings

| **Local x Global complexity** | **Mean** | **Median** | **Standard deviation** |
| --- | --- | --- | --- |
| L0xG0 | 3.42 | 3.5 | 1.43 |
| L0xG1 | 4.17 | 4.5 | 1.4 |
| L0xG2 | 4.24 | 4.5 | 1.34 |
| L1xG0 | 4 | 4 | 1.38 |
| L1xG1 | 4.07 | 4.25 | 1.26 |
| L1xG2 | 4 | 4 | 1.19 |
| L2xG0 | 3.88 | 4 | 1.3 |
| L2xG1 | 3.99 | 4 | 1.21 |
| L2xG2 | 3.94 | 4 | 1.25 |

**Supplementary Table 2. Descriptives of Arousal Ratings.**

1. Shapiro-Wilk test results of Arousal Ratings

| **Local x Global complexity** | **Shapiro-Wilk W** | **Shapiro-Wilk p** |
| --- | --- | --- |
| L0xG0 | 0.974 | <.001 |
| L0xG1 | 0.971 | <.001 |
| L0xG2 | 0.965 | <.001 |
| L1xG0 | 0.972 | <.001 |
| L1xG1 | 0.973 | <.001 |
| L1xG2 | 0.974 | <.001 |
| L2xG0 | 0.975 | <.001 |
| L2xG1 | 0.979 | <.001 |
| L2xG2 | 0.975 | <.001 |

1. Skewness and Kurtosis of Arousal Ratings

| **Local x Global complexity** | **Skewness** | **Std. error skewness** | **Kurtosis** | **Std. error kurtosis** |
| --- | --- | --- | --- | --- |
| L0xG0 | -0.185 | 0.102 | -0.187 | 0.203 |
| L0xG1 | 0.162 | 0.102 | 0.533 | 0.203 |
| L0xG2 | 0.123 | 0.102 | 0.65 | 0.203 |
| L1xG0 | 0.0612 | 0.102 | 0.369 | 0.203 |
| L1xG1 | 0.151 | 0.102 | 0.932 | 0.203 |
| L1xG2 | 0.19 | 0.102 | 0.71 | 0.203 |
| L2xG0 | 0.068 | 0.102 | 0.326 | 0.203 |
| L2xG1 | 0.172 | 0.102 | 0.678 | 0.203 |
| L2xG2 | 0.154 | 0.102 | 0.676 | 0.203 |

1. Mean and Median of Arousal Ratings

| **Local x Global complexity** | **Mean** | **Median** | **Standard deviation** |
| --- | --- | --- | --- |
| L0xG0 | 5.54 | 5.5 | 1.88 |
| L0xG1 | 5.03 | 5 | 1.51 |
| L0xG2 | 5.07 | 5 | 1.51 |
| L1xG0 | 5.11 | 5 | 1.59 |
| L1xG1 | 5.09 | 5 | 1.39 |
| L1xG2 | 5.04 | 5 | 1.42 |
| L2xG0 | 5.07 | 5 | 1.56 |
| L2xG1 | 5.07 | 5 | 1.43 |
| L2xG2 | 5.04 | 5 | 1.49 |

**Supplementary Table 3. Summary of ANOVA Results for Valence Ratings**

**
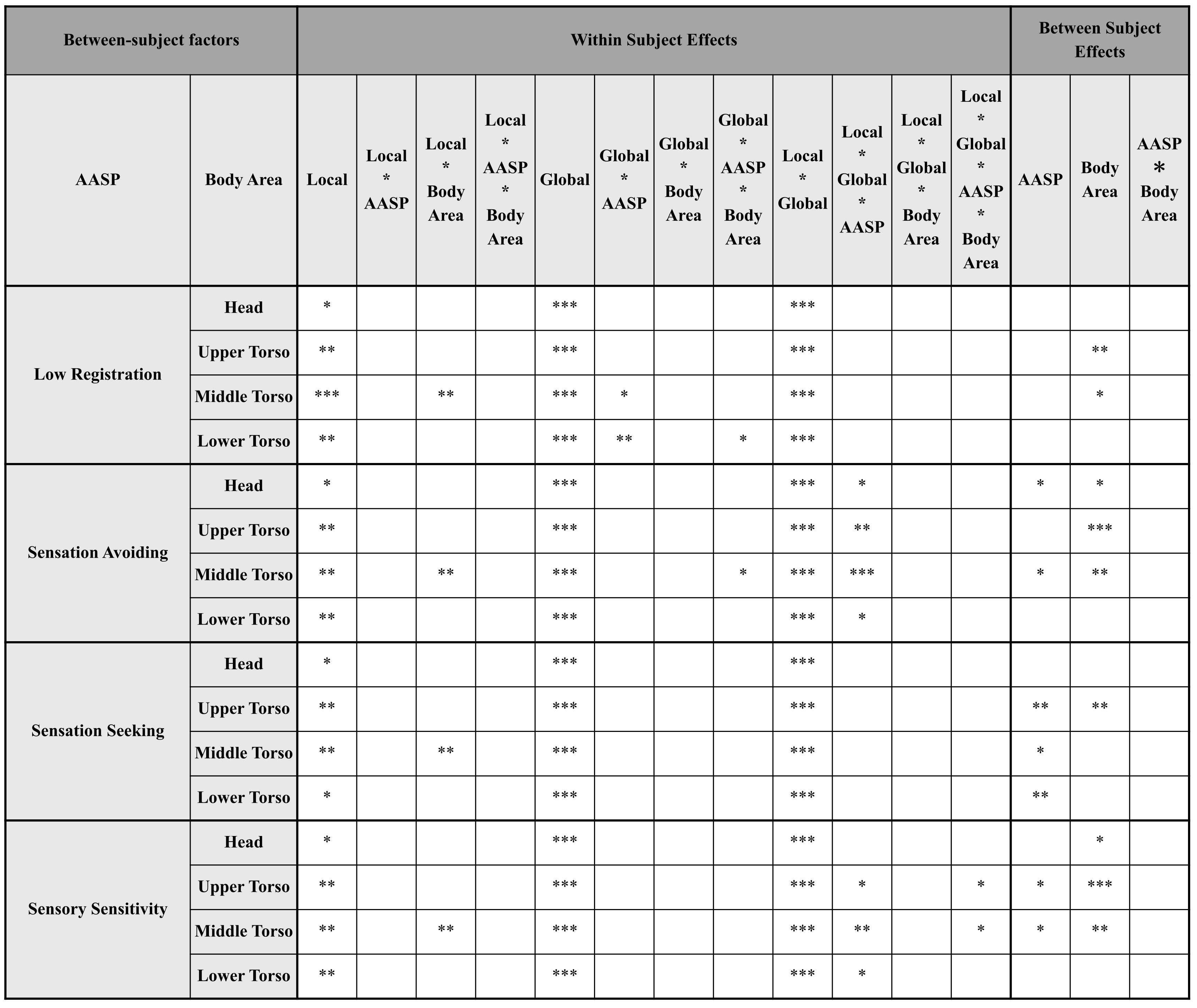
**

* p < 0.05, ** p < 0.01, *** p < 0.001

Abbreviations used in the table:
1. **AASP** - AASP sensory processing pattern 2. **Body Area** - Bodily sensation area
3. **Local** - Local Complexity 4. **Global** – Global Complexity

**Supplementary Table 4. Summary of ANOVA Results for Arousal Ratings**

**
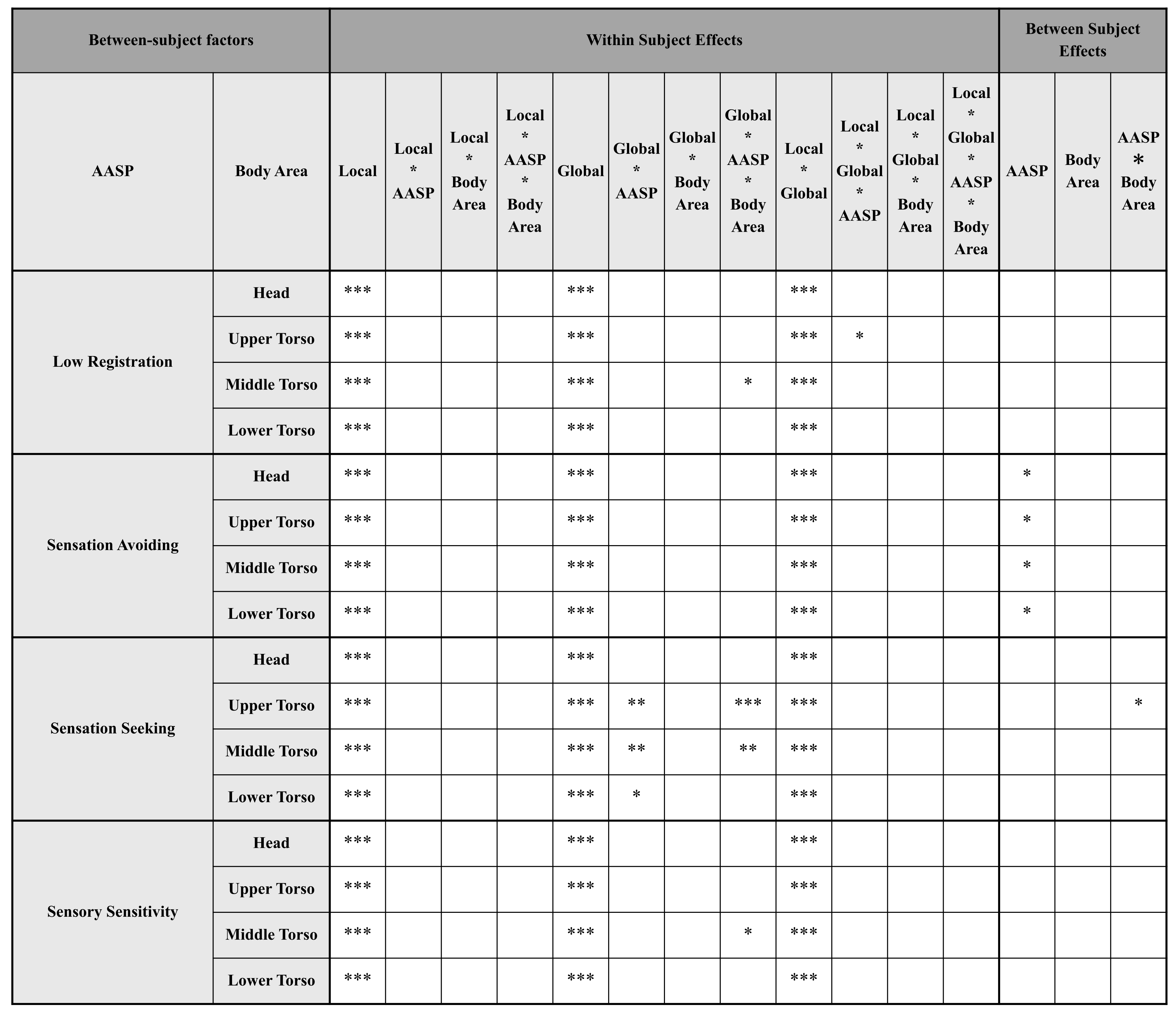
**

* p < 0.05, ** p < 0.01, *** p < 0.001

Abbreviations used in the table:
1. **AASP** - AASP sensory processing pattern 2. **Body Area** - Bodily sensation area
3. **Local** - Local Complexity 4. **Global** – Global Complexity

# Supplementary Figures


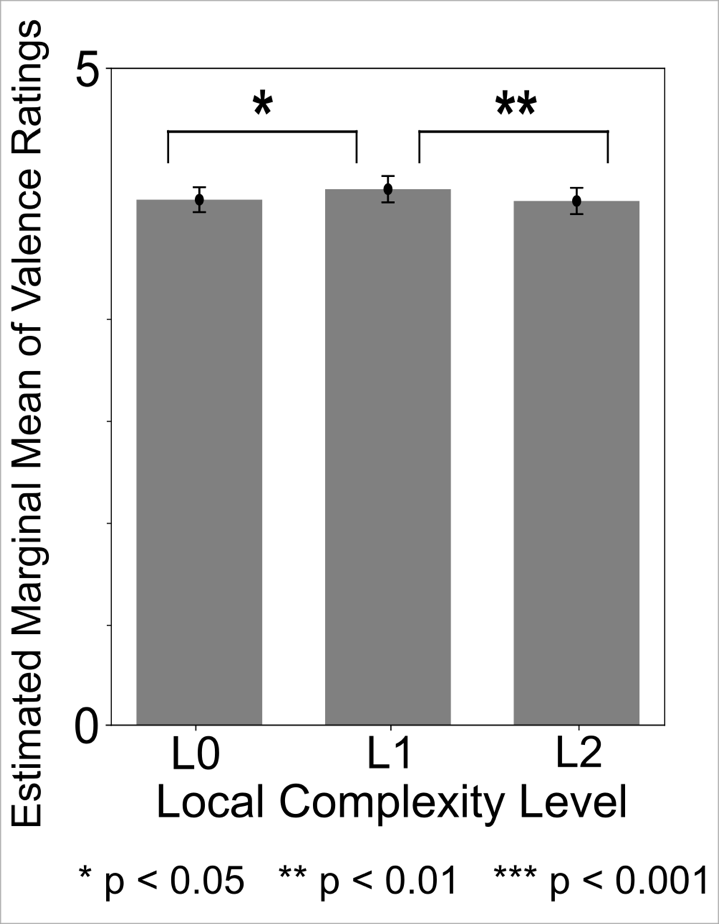


**Supplementary Figure 1. Valence Ratings by Local Complexity Level.** Valence ratings of sound sequences with Local Complexity Level of L1 (medium) were significantly higher than those of L2 (high), regardless of participants’ sensory processing patterns or bodily sensations. In some cases, including the Sensory Sensitivity and Upper Torso combination shown in this figure, valence ratings for L1 (medium) were also significantly higher than those for L0 (low).


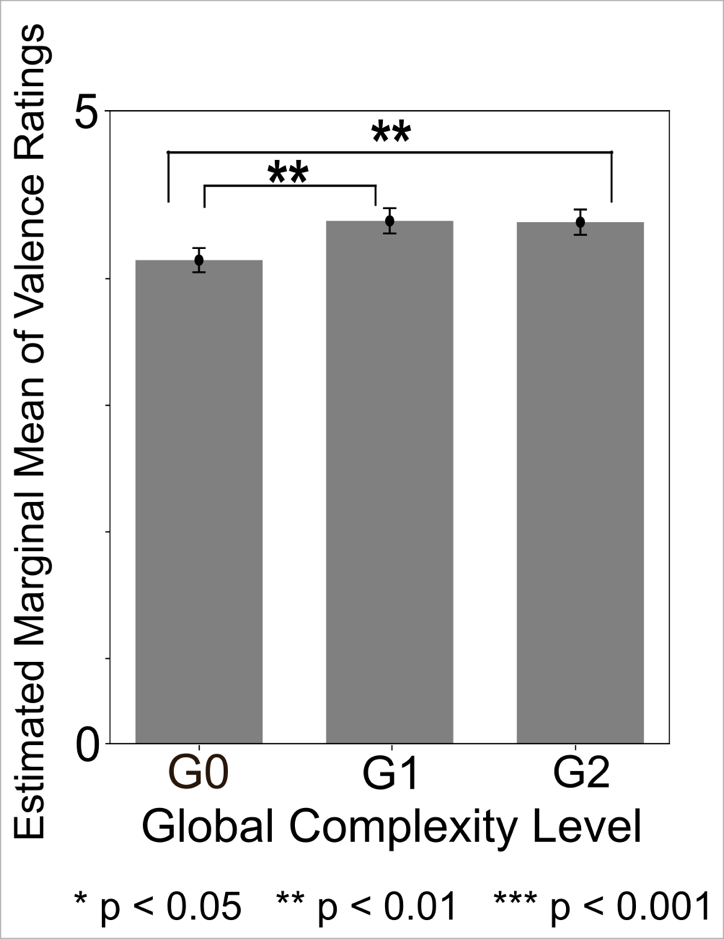


**Supplementary Figure 2. Valence Ratings by Global Complexity Level.** Valence ratings of sound sequences with Global Complexity Level of L0 (low complexity) were lower than those of both G1 and G2 (medium and high complexity), regardless of participants’ sensory processing patterns or bodily sensations.

**
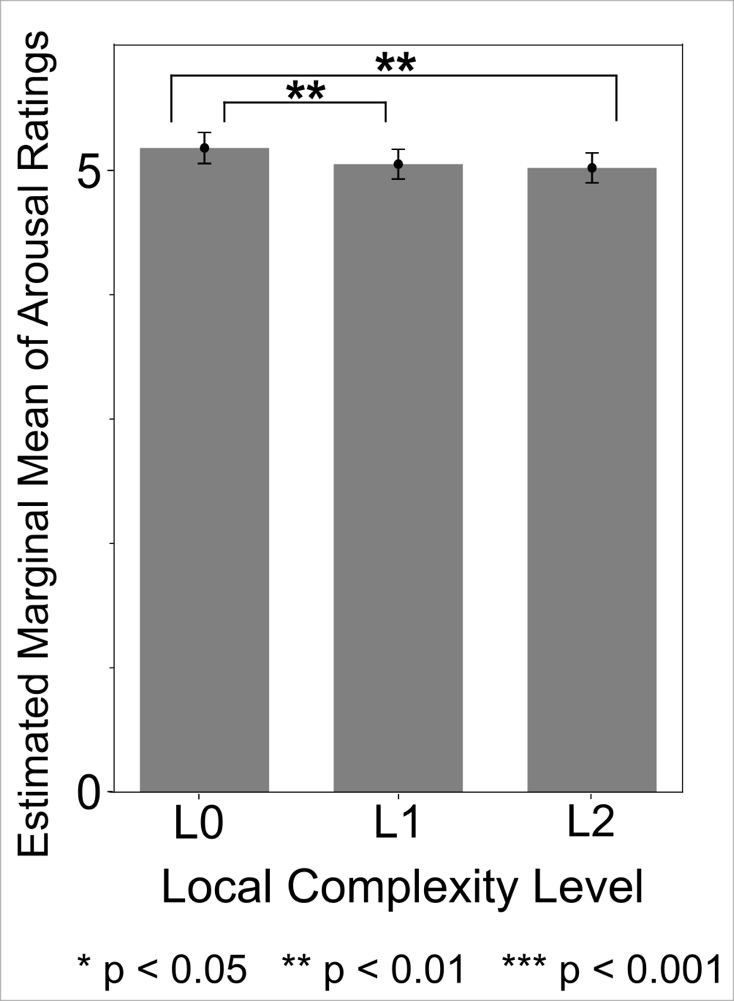
**

**Supplementary Figure 3. Arousal Ratings by Local Complexity Level.** Arousal ratings were highest for L0 sound sequences (lowest Local Complexity Level), regardless of participants’ sensory processing patterns or bodily sensations.


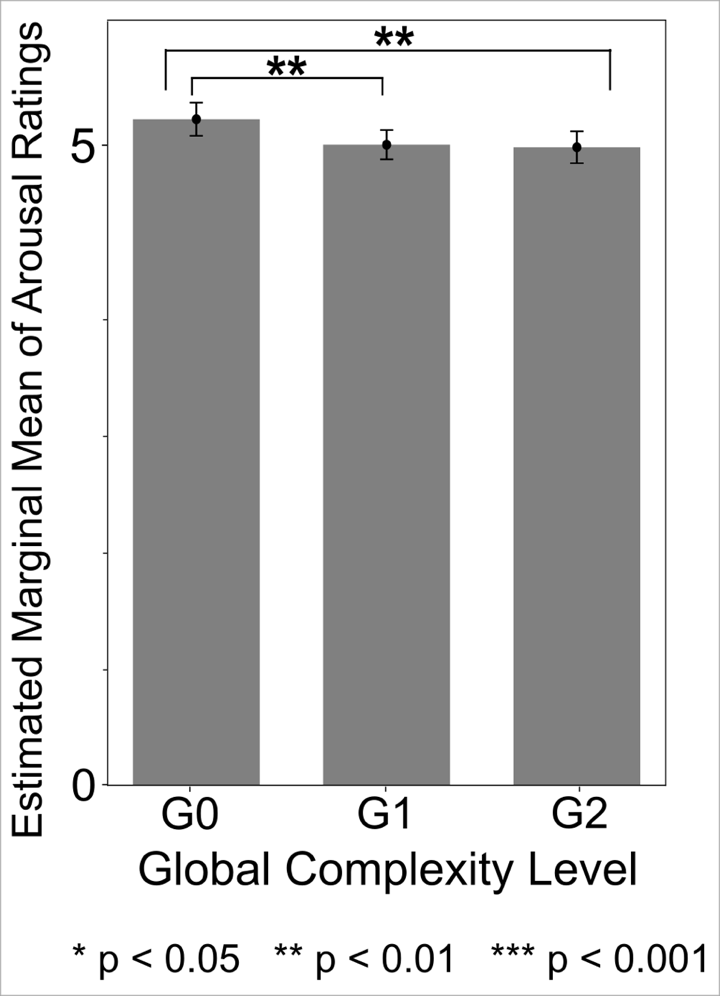


**Supplementary Figure 4. Arousal Ratings by Global Complexity Level. A**rousal ratings were highest for G0 sound sequences (lowest Global Complexity Level), regardless of participants’ sensory processing patterns or bodily sensations.

**
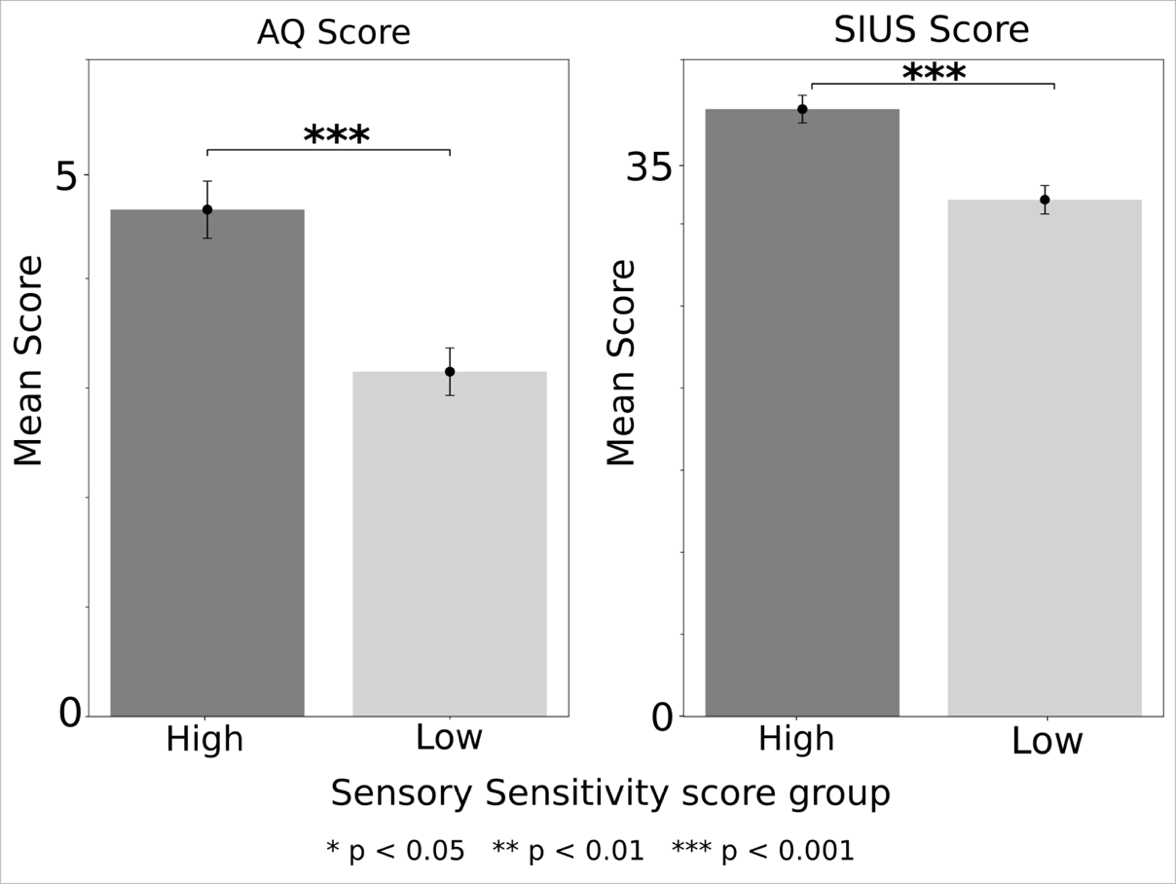
**

**Supplementary Figure 5. Differences in AQ and SIUS Mean Scores by Sensory Sensitivity Score Group.** Participants with high Sensory Sensitivity scores had significantly higher AQ and SIUS scores than those with low Sensory Sensitivity scores.

**
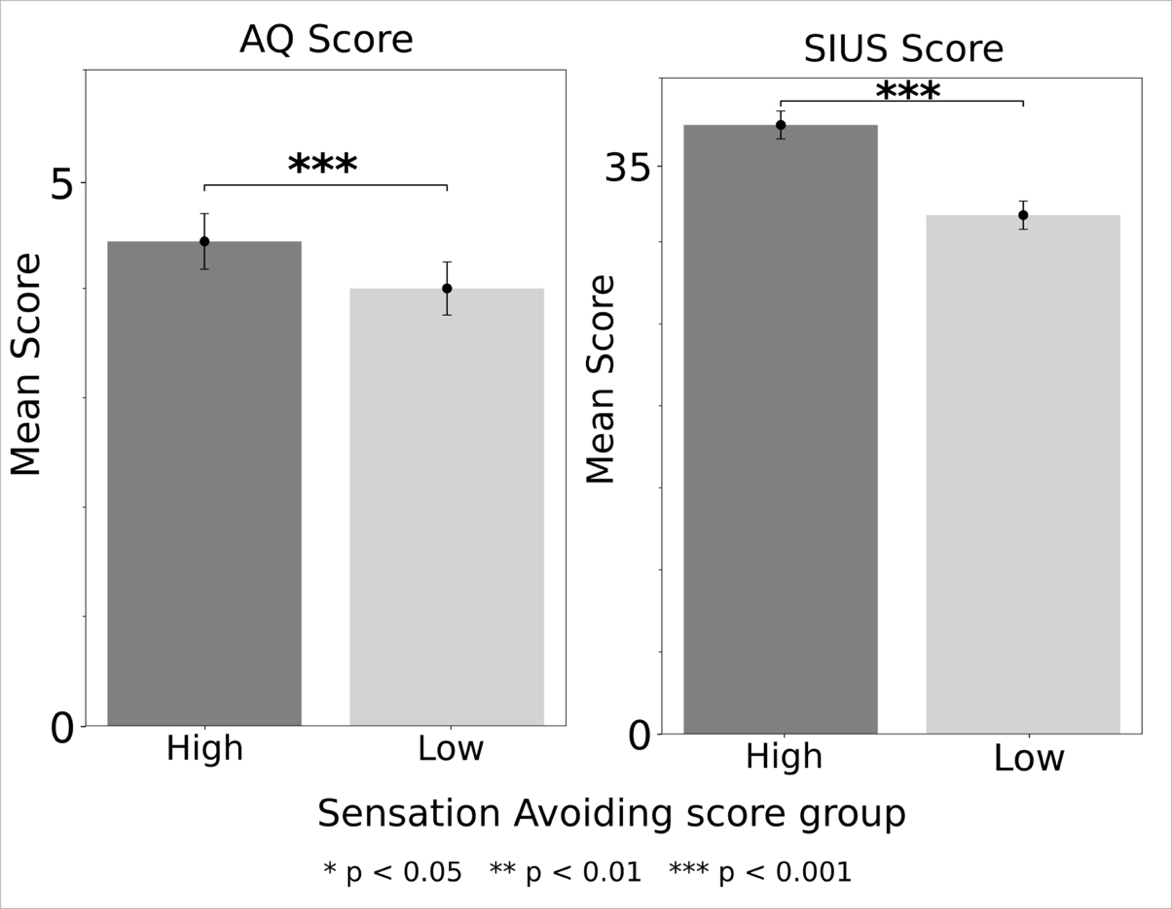
**

**Supplementary Figure 6. Differences in AQ and SIUS Mean Scores by Sensation Avoiding Score Group.** Participants with high Sensation Avoiding scores had significantly higher AQ and SIUS scores than those with low Sensation Avoiding scores.

**
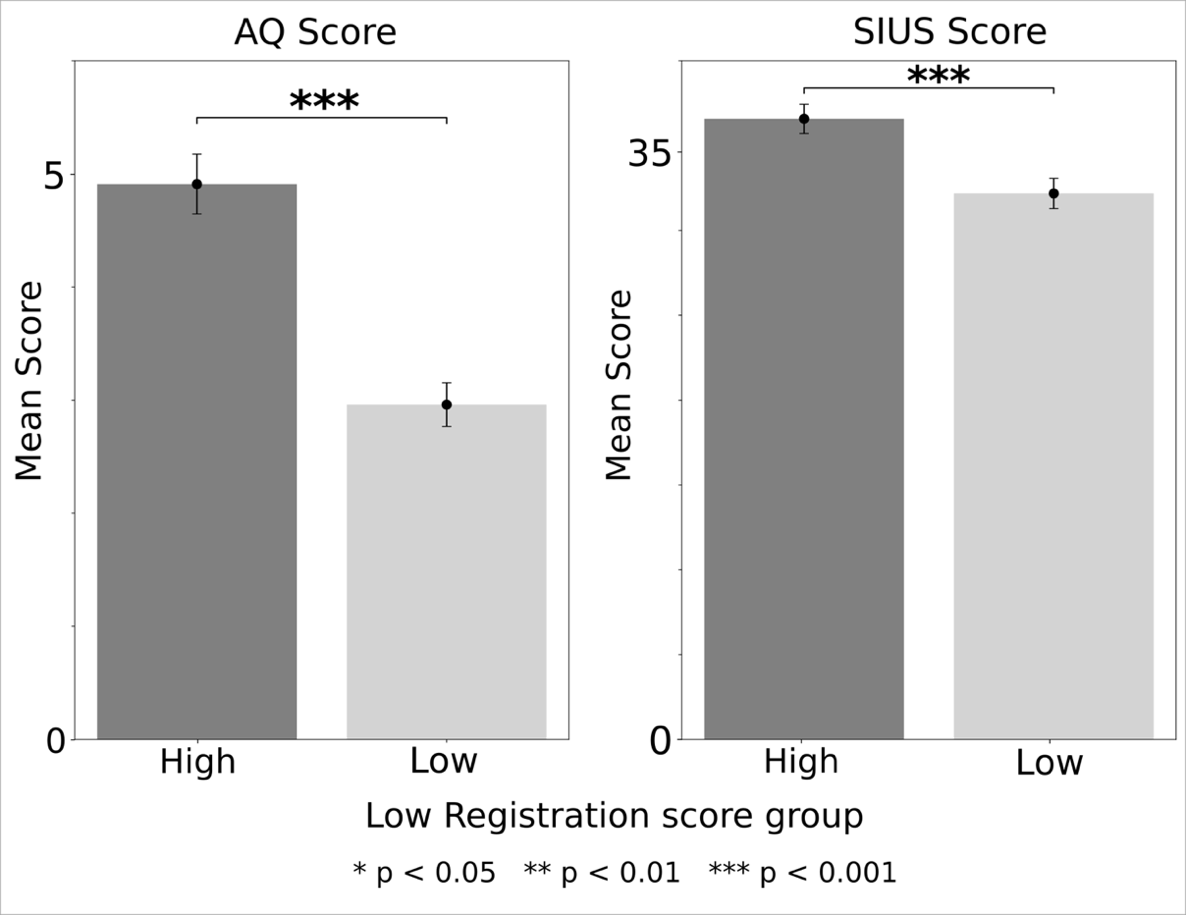
**

**Supplementary Figure 7. Differences in AQ and SIUS Mean Scores by Low Registration Score Group.** Participants with high Low Registration scores had significantly higher AQ and SIUS scores than those with low Low Registration scores.

**
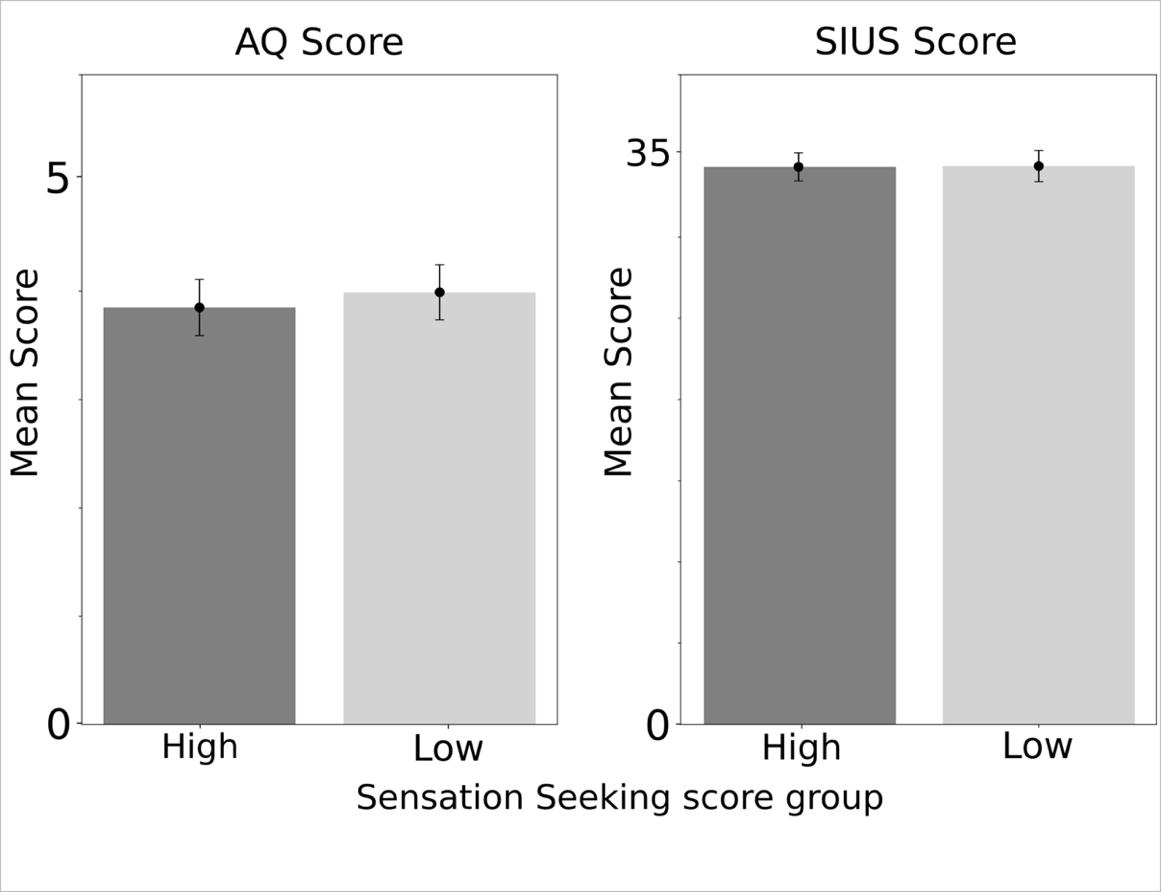
**

**Supplementary Figure 8. Differences in AQ and SIUS Mean Scores by Sensation Seeking Score Group.** No significant differences were found between AQ or SIUS scores for participants with high versus low Sensation Seeking scores.
